# Endothelial cell tropism is a determinant of H5N1 pathogenesis in mammalian species

**DOI:** 10.1371/journal.ppat.1006270

**Published:** 2017-03-10

**Authors:** Smanla Tundup, Matheswaran Kandasamy, Jasmine T. Perez, Nacho Mena, John Steel, Tamas Nagy, Randy A. Albrecht, Balaji Manicassamy

**Affiliations:** 1 Department of Microbiology, University of Chicago, Chicago, IL, United States of America; 2 Howard Taylor Ricketts Laboratory, University of Chicago, Argonne, IL, United States of America; 3 Department of Microbiology, Icahn School of Medicine at Mount Sinai, New York, NY, United States of America; 4 Department of Microbiology, Emory University, Atlanta, GA, United States of America; 5 Comparative Pathology Laboratory, University of Georgia, Athens, GA, United States of America; University of Rochester Medical Center, UNITED STATES

## Abstract

The cellular and molecular mechanisms underpinning the unusually high virulence of highly pathogenic avian influenza H5N1 viruses in mammalian species remains unknown. Here, we investigated if the cell tropism of H5N1 virus is a determinant of enhanced virulence in mammalian species. We engineered H5N1 viruses with restricted cell tropism through the exploitation of cell type-specific microRNA expression by incorporating microRNA target sites into the viral genome. Restriction of H5N1 replication in endothelial cells via miR-126 ameliorated disease symptoms, prevented systemic viral spread and limited mortality, despite showing similar levels of peak viral replication in the lungs as compared to control virus-infected mice. Similarly, restriction of H5N1 replication in endothelial cells resulted in ameliorated disease symptoms and decreased viral spread in ferrets. Our studies demonstrate that H5N1 infection of endothelial cells results in excessive production of cytokines and reduces endothelial barrier integrity in the lungs, which culminates in vascular leakage and viral pneumonia. Importantly, our studies suggest a need for a combinational therapy that targets viral components, suppresses host immune responses, and improves endothelial barrier integrity for the treatment of highly pathogenic H5N1 virus infections.

## Author summary

In healthy individuals, the symptoms of seasonal influenza virus infection are mild and the infection is cleared within 4–7 days. However, infection with highly pathogenic avian influenza virus (H5N1) can be severe and often results in fatal pneumonia even in healthy adults. While it is known that both viral and host factors play a role in enhanced disease progression, the molecular mechanisms for the high virulence of H5N1 virus are not completely understood. In this study, we engineered avian influenza H5N1 viruses incapable of replicating in endothelial cells and evaluated disease symptoms in mice and ferrets. Our studies show that H5N1 infection of endothelial cells causes severe disease and death of infected animals in part due to the damage of endothelial cells lining the blood vessels, which results in leakage of fluid into the lungs (pneumonia).

## Introduction

Influenza A viruses, members of the *Orthomyxoviridae* family, pose a constant threat to human health with seasonal epidemics and occasional pandemics. It is estimated that seasonal influenza virus infections result in 250,000–500,000 annual deaths worldwide [1]. Seasonal influenza virus infections in healthy adults are self-limiting and are primarily restricted to the upper respiratory tract; however, infections in children and the elderly are potentially severe and can result in viral pneumonia. In addition to humans, influenza A viruses can infect a wide range of host species including waterfowl, swine, domestic birds, and seals. As such, influenza A viruses circulating in zoonotic reservoirs have intermittently caused widespread infections and even pandemics in humans [2,3]. The last four influenza pandemics—1918 H1N1 Spanish flu, 1957 H2N2 Asian flu, 1968 H3N2 Hong Kong flu, and 2009 H1N1—involved influenza A virus transmission from zoonotic reservoirs into humans [3,4,5]. Moreover, influenza A virus strains such as H5N1, H7N7, and H7N9 have crossed the species barrier from domestic poultry to cause fatal infections in humans [6,7]. Fortunately, these avian viruses are incapable of causing sustained human-to-human transmission and thus the majority of these infections have been the result of direct contact with infected poultry; however, recent studies indicate that less than 5 mutations are sufficient to render avian H5N1 viruses transmissible via respiratory droplets [8,9]. Thus, a better understanding of the pathogenic nature of avian influenza A viruses is important for devising novel therapeutic strategies and preventing future pandemics.

As compared to seasonal influenza A virus infections, infections with highly pathogenic avian influenza (HPAI) H5N1 virus result in fatal viral pneumonia across all age groups including healthy adults [10,11,12]. Histopathological analyses of fatal human H5N1 infections have demonstrated extensive alveolar damage in the lower respiratory tract [13,14,15,16]. This is in part due to increased infiltration of inflammatory cells and elevated levels of proinflammatory cytokines, implicating the overt activation of host immune responses in the disease progression and immunopathology of H5N1 infections [13]. This immunopathology has been recapitulated in animal models, as infected animals show excessive infiltration of inflammatory monocytes and neutrophils, and hypercytokinemia in the lungs [17,18,19]. While it has been well appreciated that epithelial cells and innate immune cells are the major source of cytokines during influenza A virus infection, recent work with a mouse adapted laboratory strain indicates that endothelial cells are also a source of proinflammatory cytokines in the lungs [17,20,21]. Thus, it has been proposed that influenza A virus pathogenesis is the result of endothelial cell activation by proinflammatory cytokines in the lungs, as well as endothelial damage due to excessive infiltration of leukocytes that increases endothelial barrier permeability, culminating in viral pneumonia [22,23]. Interestingly, in avian hosts, high levels of HPAI virus infection of endothelial cells causes vascular leakage and widespread hemorrhaging [24,25]. However, there are limited reports on infection of endothelial cells in humans or mammalian animal models [26]. As in vitro studies demonstrate that human endothelial cells are capable of supporting HPAI virus replication, it remains to be determined if HPAI virus infection of endothelial cells contributes to pathogenesis in mammalian species.

Here, we investigated the importance of endothelial cell tropism in the virulence and pathogenesis of HPAI virus infection in mouse and ferret models. To this end, we engineered H5N1 viruses with restricted cell tropism through the incorporation of microRNA (miRNA) target sites into the 3’ untranslated region (3’UTR) of the viral NP segment as previously described [27,28,29]. Specifically, we generated H5N1 viruses containing endothelial cell specific miR-126 target sites (H5N1-126T) or hematopoietic cell specific miR-142 target sites (H5N1-142T), such that viral replication was abrogated in endothelial cells or hematopoietic cells, respectively. Mice infected with H5N1-126T virus showed (1) greatly ameliorated disease symptoms, (2) decreased levels of proinflammatory cytokines, (3) limited vascular leakage, and (4) restriction of replication to the lungs, despite demonstrating similar peak viral titers in the lungs as compared to a scrambled control virus (H5N1-ScrbT). In agreement with these results, ferrets infected with H5N1-126T virus showed no clinical symptoms of HPAIV infection such as weight loss or neurological symptoms, and displayed decreased viral spread to the lower respiratory tract, olfactory bulb, and brain, despite displaying similar viral titers in the nasal washes in comparison to the control H5N1-ScrbT virus. In contrast, restriction of viral replication in hematopoietic cells via miR-142 did not abrogate H5N1 infection-induced pathogenesis in mice, despite demonstrating decreased proinflammatory cytokine production in the lungs as compared to H5N1-ScrbT. Taken together, our studies demonstrate that the endothelial cell tropism of H5N1 virus is an important determinant of virulence and pathogenesis in mammalian species.

## Results

### Endogenous miRNA mediated restriction of H5N1 cell tropism

**Fig 1:**
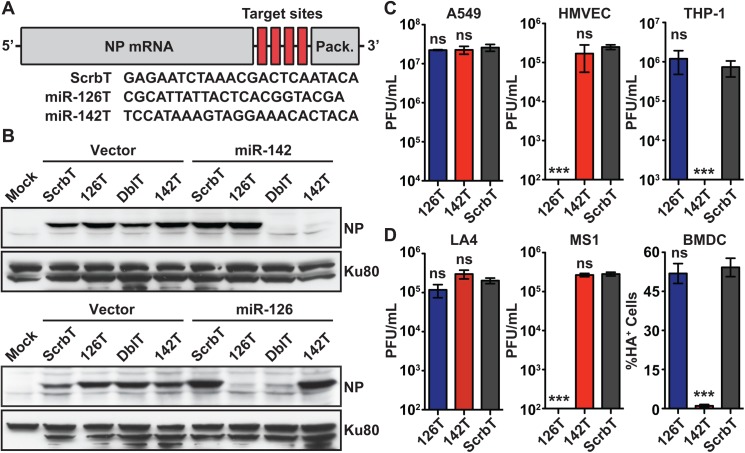
Generation and characterization of H5N1 viruses with restricted tropism. (A) Schematic representation of NP mRNA carrying four copies of miRNA target sites in the 3’UTR. The incorporated target sequences, which are Complementary to the mature miRNA, are shown below. (B) Evaluation of miRNA mediated restriction of NP expression. HEK-293T cells were co-transfected with plasmids expressing specific miRNAs (vector, miR142, miR126) and NP with miRNA target sites, and NP expression was analyzed by western blot. DblT refers to the NP segment carrying two miR-142-3p and two miR-126-3p target sites. Expression of Ku80 is shown as a loading control. (C-D) Evaluation of tropism restricted H5N1 virus replication in human and murine cells. (C) Human epithelial cells (A549, MOI = 0.001), endothelial cells (HMVEC, MOI = 0.01), monocytic leukemia cells (THP-1, MOI = 3), and (D) mouse epithelial cells (LA-4; MOI = 1), endothelial cells (MS1, MOI = 1) and dendritic cells (BMDC, MOI = 0.1) were infected with tropism restricted H5N1 viruses and viral titers at 48 hrs pi (hpi) were determined by plaque assay for all but BMDC. The titers are shown as PFU/mL (mean ± SEM). The limit of detection is 10 PFU/mL. For BMDC, the level of infection at 24hpi was measured by flow cytometry using an anti-H5 antibody. Data is represented as percentage of HA^+^ cells in the CD11c^+^ population (mean ± SEM). Asterisk denotes statistical significance determined by one-way ANOVA in comparison to H5N1-ScrbT group and the values are denoted as *p<0.05, **p<0.01, ***p<0.001 and ns–non significant. Data presented here is a representative of at least three independent experiments performed in triplicate.

To investigate if the enhanced virulence associated with H5N1 infection is due to broad cell tropism, we engineered H5N1 viruses (A/Vietnam/1203/2004) with specifically restricted cell tropism based on the strategy developed by the tenOever group [27,28,29]. To generate H5N1 viruses incapable of replicating exclusively in hematopoietic or endothelial cells, we incorporated four copies of miRNA target sites (complementary sequence of a miRNA) for miR-142-3p (142T) or miR-126-3p (126T), respectively, into the 3’ UTR of the viral NP segment (Fig 1A). Prior miRNA profiling studies have shown that miR-142 and miR-126 are specifically expressed in hematopoietic or endothelial cells, respectively [27,28,30,31]. We also engineered an NP segment carrying two miR-142-3p and two miR-126-3p target sites (DblT) as well as a control NP segment carrying a scrambled target sequence (ScrbT, Fig 1A). To determine if the engineered NP segment was sensitive to specific miRNA expression, we evaluated NP protein levels in HEK293 cells during coexpression of individual miRNAs by western blot analysis. Expression of miR-142 specifically suppressed protein production from NP segments carrying the corresponding target site (142T and DblT) but had no effect on ScrbT or 126T; similarly, expression of miR-126 suppressed NP protein production from 126T and DblT (Fig 1B) [32]. Next, we generated H5N1 viruses carrying these miRNA targeted NP segments (H5N1-142T, H5N1-126T, and H5N1-ScrbT) and examined replication in human and mouse cells. In our preliminary studies, H5N1-DblT showed intermediate levels of replication and thus was removed from further analysis. In human lung epithelial cells (A549), all three viruses replicated to similar levels, with a peak titer of 10^7^ plaque forming units/milliliter (PFU/mL) (Fig 1C; S1 Fig). In addition, the replication kinetics of H5N1-ScrbT were similar to recombinant wild-type H5N1 virus (H5N1-WT) in all cell types, demonstrating that incorporation of scrambled miRNA target sites into the 3’UTR of NP did not significantly alter H5N1 replication (S1 Fig). As anticipated, replication of H5N1-126T was completely abrogated in primary human microvascular endothelial cells (HMVEC), while replication of H5N1-142T and H5N1-ScrbT was uninhibited (Fig 1C; S1 Fig). Similarly, only replication of H5N1-142T was inhibited in human leukemia monocytic cells (THP-1, Fig 1C; S1 Fig). As these miRNAs are highly conserved across species, we evaluated the replication of miRNA-targeted viruses in mouse cell lines (S1 Table; Fig 1D and S1 Fig). In agreement with our observations in human cells, all three viruses replicated to equivalent levels in mouse lung epithelial cells (LA4), and replication of H5N1-126T was inhibited in murine endothelial cells (MS1, Fig 1D; S1 Fig). Furthermore, replication of H5N1-142T was suppressed in mouse bone marrow derived dendritic cells (BMDC) and a macrophage like cell line (J774; Fig 1D and S1 Fig), both of which express high levels of miR-142 (Fig 1D) [28,31]. Taken together, these results demonstrate that endogenous cell type specific miRNAs can be utilized to restrict H5N1 cell tropism.

### Restriction of H5N1 replication in endothelial cells ameliorates morbidity and mortality in mice

**Fig 2:**
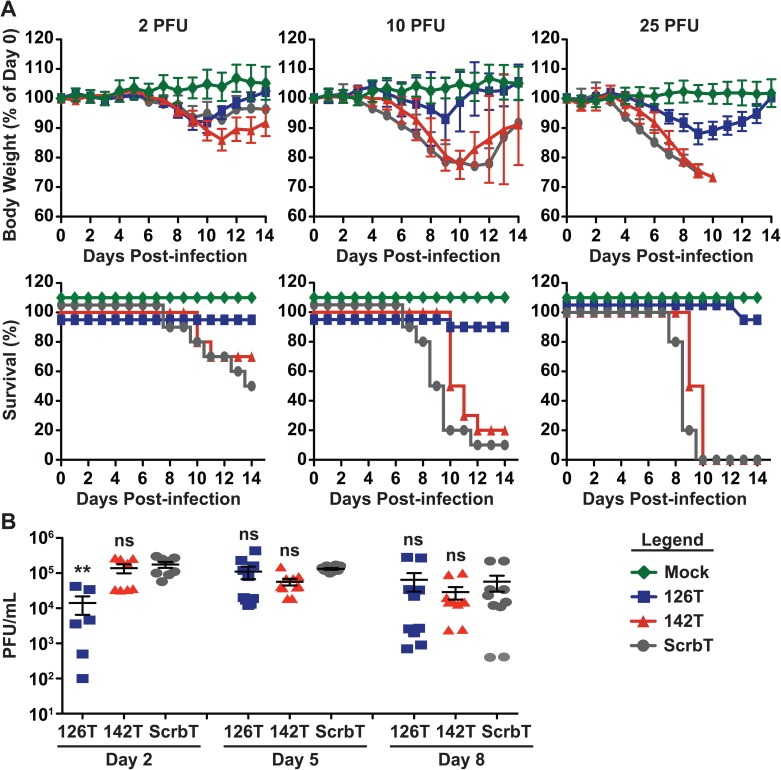
Restriction of H5N1 replication in endothelial cells ameliorates disease symptoms in mice. (A) C57BL/6J mice (n = 10) were intranasally infected with different doses (2, 10, and 25 PFU) of tropism restricted H5N1 viruses and monitored daily for body weight and survival. Top—Body weight loss, shown as relative percentage of day 0 weight (mean ± SEM) and Bottom–Survival. For better display, survival lines are offset by a few percentage points. (B) Viral titers in the lungs of infected mice. C57BL/6J mice (n = 6–10) were infected with 25 PFU and viral loads in the lungs on days 2, 5, and 8 pi were determined by plaque assay (PFU/mL). Each data point represents an individual mouse (mean ± SEM). Asterisk denotes statistical significance determined by one-way ANOVA in comparison to H5N1-ScrbT group and the values are denoted as *p<0.05, **p<0.01, ***p<0.001 and ns–non significant. Data presented here is a pooled average of two independent experiments.

To determine if endothelial cell or hematopoietic cell tropism contributes to virulence, C57BL/6J mice were intranasally infected with different doses (2, 10 and 25 PFU) of the H5N1 viruses, and monitored for weight loss and survival for 14 days. Mice infected with H5N1-ScrbT showed pronounced weight loss (>25%) and the majority of mice succumbed to infection at both the 10 and 25 PFU dose (Fig 2A). In contrast, the H5N1-126T infected group displayed reduced weight loss (~10–15%) at the highest dose (25 PFU) and all but one mouse survived the infection (Fig 2A). H5N1-142T infected mice showed weight loss and survival similar to the H5N1-ScrbT group (Fig 2A). In our control studies with H5N1-ScrbT and H5N1-WT, we did not observe significant differences in the disease symptoms in mice, indicating that insertion of miR ScrbT sites did not alter the virulence of H5N1 virus in mice (S2 Fig). Next, we examined if the altered pathogenicity observed in H5N1-126T infected mice was due to differences in viral replication in the lungs. Although viral titers in the lungs of H5N1-126T infected mice were reduced on day 2 post-infection (pi), by day 5 and day 8 pi there was no discernible difference in titers for all three groups (Fig 2B). Taken together, these results demonstrate that endothelial cell restricted H5N1-126T demonstrated reduced pathogenicity in mice, despite replicating at levels similar to control H5N1-ScrbT and hematopoetic restricted H5N1-142T.

### H5N1 infection of the endothelial and hematopoietic compartments contributes to hypercytokinemia

**Fig 3:**
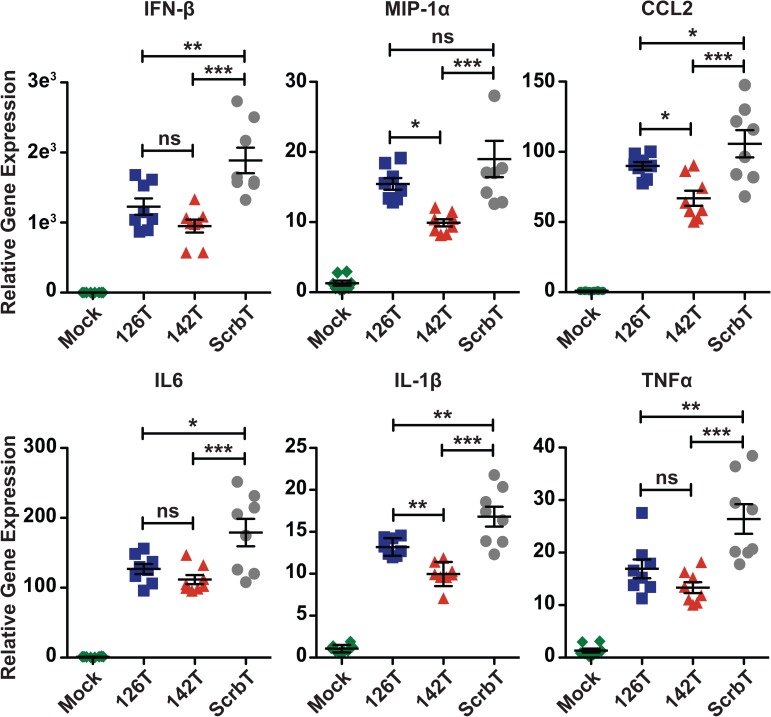
Restriction of cell tropism alters cytokine and chemokine responses in the lungs. C57BL/6J mice (n = 8) were intranasally infected with a 25 PFU dose of the H5N1 viruses and total RNA from the lungs was isolated on day 5 pi via Trizol extraction. Expression levels of inflammatory genes (IFN-β, MIP-1α, CCL2, IL-6, IL-1β and TNFα) were measured by quantitative PCR analyses. Data is represented as fold expression relative to mock infected mice (mean ± SEM). Asterisk denotes statistical significance determined by one-way ANOVA and the values are denoted as *p<0.05, **p<0.01, ***p<0.001 and ns–non significant. Data presented here is a representative of at least two independent experiments.

A prior study using WSN/33 (a mouse adapted strain) indicated that endothelial cells contribute to excessive production of inflammatory cytokines [20]. In addition, infections with highly virulent influenza virus strains are associated with excessive production of proinflammatory cytokines [13,33,34]. To determine if the differences in the pathogenesis of H5N1 viruses with restricted cell tropism can be attributed to disparate levels of inflammatory cytokines in the lungs, we performed quantitative PCR (qPCR) analysis of inflammatory gene expression in lung homogenates of H5N1 virus infected mice. qPCR analysis indicated significantly higher levels of proinflammatory cytokines and chemokines in the lungs of H5N1-ScrbT infected mice as compared to the other two groups (Fig 3). The lowered levels of cytokines in the lungs of H5N1-142T and H5N1-126T infected mice suggests that infection and activation of both hematopoietic and endothelial cells contributes to hypercytokinemia during H5N1 infection. Interestingly, the H5N1-142T infected group displayed lower levels of some pro-inflammatory cytokines as compared to the H5N1-126T group; however, H5N1-142T infected mice succumbed to infection whereas the majority of the H5N1-126T infected group survived (Fig 3 and Fig 2A). These data show that elevated levels of cytokines are not solely responsible for the severe pathogenesis of H5N1 virus.

### Restriction of H5N1 replication in endothelial cells reduces microvascular leakage in the lungs

**Fig 4:**
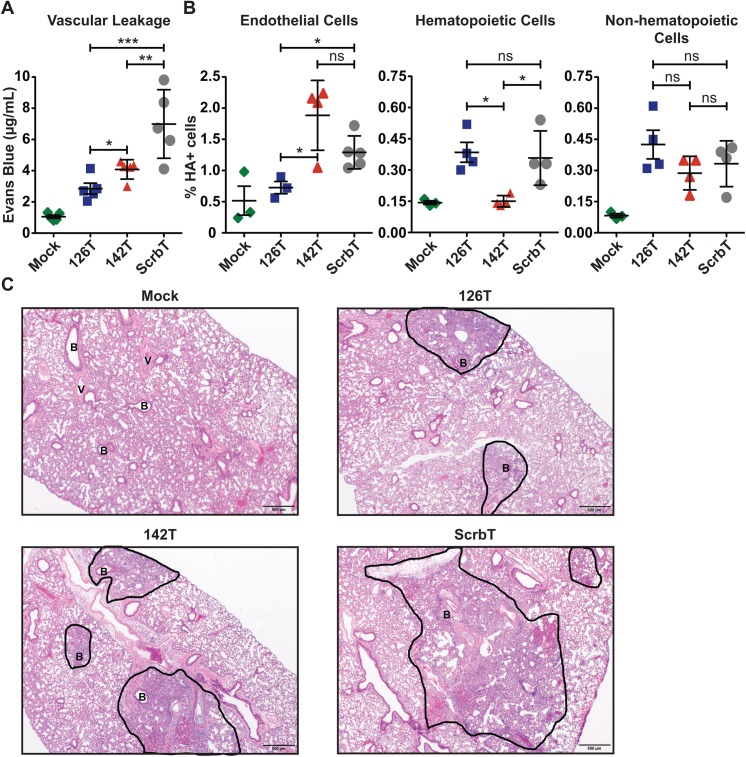
H5N1 infection of endothelial cells increases vascular leakage and causes pronounced damage in the lungs. (A) Evaluation of vascular leakage in H5N1 infected lungs. C57BL/6J mice (n = 5) were intranasally infected with 25 PFU of the H5N1 viruses. On day 7 pi, Evans Blue dye was injected into mice via retro-orbital route. After 1h, mice were euthanized and the levels of Evans Blue dye in the BALF were measured. Each data point represents the concentration of Evans Blue dye in the BALF of individual mice (mean ± SEM). (B) Evaluation of infection of various cell populations in vivo. C57BL/6J mice (n = 3) were infected with 25 PFU of the H5N1 viruses. On day 3 pi, lungs were harvested and analyzed for H5N1 infection of the various cell compartments by flow cytometry. Cell surface markers CD45 and CD31 were used to distinguish endothelial cells (CD45^-^, CD31^+^), hematopoietic cells (CD45^+^) and non-hematopoietic cells (CD45^-^), and surface expression of viral HA was used to define infected cells (HA^+^). Data is represented as the mean (± SEM). Asterisk denotes statistical significance determined by one-way ANOVA and the values are denoted as *p<0.05, **p<0.01, ***p<0.001 and ns–non significant. Data presented here is a representative of at least two independent experiments. (C) Histopathological analysis of murine lungs by hematoxylin and eosin staining. C57BL/6J mice (n = 5) were intranasally infected with 25 PFU of the H5N1 viruses and on day 5 post-infection, the lungs were isolated and analyzed by H&E staining. The areas of infection and inflammation are indicated and outlined in black. B-bronchiole, V-blood vessel.

Prior studies suggest that both proinflammatory cytokines and infection of endothelial cells can increase endothelial permeability, eventually resulting in viral pneumonia [22,35,36,37,38]. As H5N1-142T infected mice displayed lower levels of proinflammatory cytokines yet succumbed to infection, we examined changes in vascular integrity and the levels of infection in the endothelial cell compartment using the Evans Blue dye test and flow cytometry, respectively [39]. Mice were intranasally infected with the H5N1 viruses and retro-orbitally injected with Evans Blue dye 1hr prior to sacrifice on day 7 pi, and the level of dye in the bronchioalveolar lavage fluid (BALF) was quantified. H5N1-126T infected mice showed significantly lower levels of Evans Blue dye in the BALF as compared to control H5N1-ScrbT infected mice, and H5N1-142T infected mice showed an intermediate phenotype, with levels of Evans Blue dye higher than observed for H5N1-126T yet lower than observed for H5N1-ScrbT (Fig 4A). As infection of endothelial cells can affect barrier function, we examined if the increased permeability in the lungs of H5N1-ScrbT and H5N1-142T infected mice was due to direct infection of endothelial cells. We observed a higher percentage of HA positive endothelial cells (CD45^-^ CD31^+^) in H5N1-ScrbT and H5N1-142T infected mice as compared to H5N1-126T infected mice (Fig 4B). As demonstrated previously by others, the percentage of HA positive hematopoietic cells (CD45^+^) was lower in H5N1-142T infected mice as compared to H5N1-ScrbT and H5N1-126T infected mice (Fig 4B) [28]. In contrast, we observed similar percentages of HA positive non-hematopoietic cells for all three groups (CD45^-^). Taken together, these results demonstrate that both the direct infection of endothelial cells as well as higher levels of proinflammatory cytokines contribute to increased vascular leakage during H5N1 infection.

To understand the pathological changes in the lung upon infection with miR-targeted viruses, we isolated lungs from infected mice and performed hematoxylin and eosin staining (H&E) analysis of lung sections (Fig 4C). Interestingly, smaller areas of infection and inflammation were observed in the lungs of H5N1-126T infected mice as compared to H5N1-ScrbT infected mice; however, the sizes of areas of inflammation in H5N1-142T infected mice were intermediate between the other two groups. The differences in the sizes of lesions among different groups of infected mice correlated well with the levels of vascular leakage observed in the lungs by Evans Blue test (Fig 4A). These results demonstrate that endothelial cell tropism of H5N1 causes increased damage in the lungs.

### Increased microvascular leakage facilitates systemic spread of H5N1 virus

**Fig 5:**
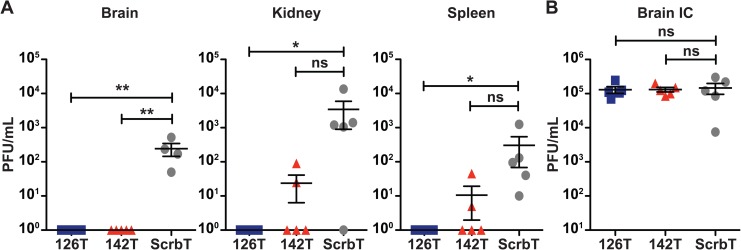
Endothelial cell infection induced damage is necessary for extrapulmonary spread of H5N1 virus. (A) Viral titers in different organs. C57BL/6J mice (n = 5) were intranasally infected with 25 PFU of the H5N1 viruses and on day 8 pi, different organs were harvested for viral titer determination. Each data point represents an individual mouse (PFU/mL). (B) Viral replication in the brain upon intracerebellar injection. C57BL/6J mice (n = 3–5) were injected with 100 PFU of the H5N1 viruses in the cerebellum and on day 3 pi whole brains were isolated for viral titer determination. Each data point represents the viral titer of an individual mouse (PFU/mL; mean ± SEM). The limit of detection is 10 PFU/mL. Asterisk denotes statistical significance determined by one-way ANOVA and the values are denoted as *p<0.05, **p<0.01, ***p<0.001 and ns–non significant. Data presented here is a representative of at least two independent experiments.

Previous studies demonstrate that the multibasic cleavage site (MBS) in HA is essential for the spread of HPAI virus infection to the extrapulmonary organs [40,41]. To determine if the systemic spread of H5N1 virus occurs via the disruption of barrier integrity and/or infection of endothelial cells, mice were intranasally infected with the H5N1 viruses and viral titers in different organs were measured on day 8 pi. In H5N1-126T infected mice, we could not detect any virus replication outside of the respiratory tract, including the brain, kidney, and spleen, despite the presence of the MBS in HA (Fig 5A). In contrast, virus replication was readily detected in the extrapulmonary organs of H5N1-ScrbT infected mice (Fig 5A). Interestingly, H5N1-142T infected mice displayed an intermediate phenotype for extrapulmonary viral spread, as virus was detected in the kidney and spleen for a portion of infected mice (Fig 5A), despite showing endothelial cell infection at levels similar to the H5N1-ScrbT group (Fig 4B). These results demonstrate that the level of extrapulmonary spread of H5N1 virus is determined by the extent of vascular damage in the lungs. To demonstrate that the lack of extrapulmonary spread for H5N1-126T was not due to an inability to replicate in other organs, we performed direct intracerebellar injection in mice and assessed viral loads on day 3 pi. All 3 H5N1 viruses replicated to equally high titers in the brains of infected mice, indicating that H5N1-126T was unable to spread to extrapulmonary organs, likely due to its restricted endothelial cell tropism (Fig 5B). These results demonstrate that, in addition to the MBS in HA, increased microvasular damage and leakage facilitates the extrapulmonary spread of H5N1 virus.

### Endothelial cell tropism contributes to H5N1 pathogenesis in the ferret model

**Fig 6:**
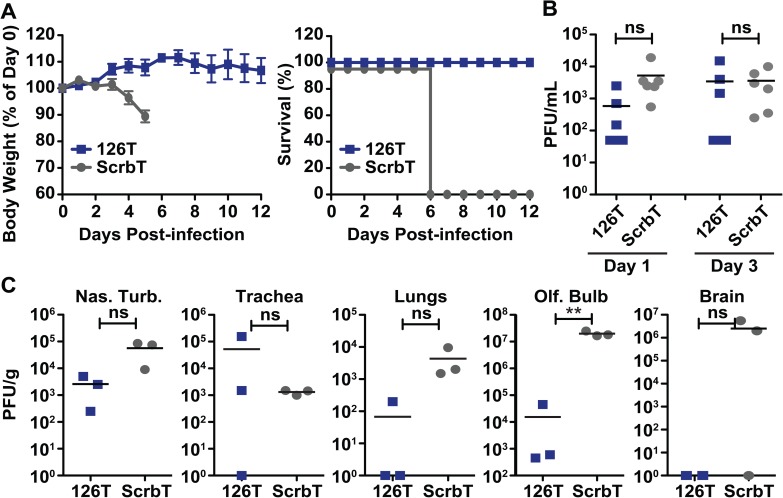
Restriction of H5N1 replication in endothelial cells ameliorates disease symptoms in ferrets. Five-month old male finch ferrets that tested seronegative for circulating influenza viruses were intranasally infected with a 3500 PFU dose (n = 6/group) of the H5N1 viruses, and monitored for weight loss and clinical signs of infection. (A) Weight loss and survival of infected ferrets. On day 5 pi, H5N1-ScrbT infected ferrets showed severe signs of neuronal infection and were humanely euthanized. (B) Viral loads in the nasal washes. Nasal washes were performed on day 1 and 3 pi and viral titers were determined by plaque assay. (C) Viral replication at different sites in the respiratory tract and brain. On day 4 pi, three ferrets from each group were euthanized and different organs were harvested for viral titer determination. Each data point represents the viral titer of an individual ferret. The limit of detection is 10 PFU/mL. Asterisk denotes statistical significance determined by one-way ANOVA and the values are denoted as *P<0.05, **P<0.01, ***P<0.001 and ns–non significant. Ferret experiment was performed once.

Ferrets are considered an excellent model to study influenza virus pathogenesis, and respiratory epithelial cells are the primary sites of influenza virus replication in ferrets [42,43]. To exclude the possibility of any differences in replication, we evaluated the replication of miR-targeted viruses and H5N1-WT in a ferret epithelial like cell line and observed similar levels of replication for all four strains (S3 Fig). As miR-126 is highly conserved across multiple species, including ferrets and chickens, we next evaluated the virulence of the generated H5N1 viruses in the ferret model (S1 Table). Ferrets were intranasally infected with the H5N1 viruses and monitored for weight loss and clinical signs of infection. H5N1-ScrbT infected ferrets began to lose weight on day 3 pi, and by day 6 pi infected ferrets showed signs of severe disease, including weight loss (>10%) and neurological complications, and were humanely euthanized (Fig 6A). In contrast, ferrets infected with H5N1-126T did not display any weight loss and survived for the duration of the experiment (12 days). Analysis of nasal washes for viral replication showed no statistically significant differences in the viral loads of both groups on day 1 and 3 pi (Fig 6B). These studies demonstrate that endothelial cell tropism is a determinant of H5N1 pathogenesis in ferrets.

### Restriction of H5N1 replication in endothelial cells reduces viral spread to the lower respiratory tract

Previous studies show that H5N1 viruses can spread to the lower respiratory tract of infected ferrets [41,44]. Our analysis of the distribution of H5N1 in various regions of the respiratory tract (day 4 pi) demonstrated a modest difference in viral loads in the nasal turbinates of H5N1-ScrbT and H5N1-126T infected ferrets; however, viral replication in the trachea was similar for both groups (Fig 6C). Interestingly, viral replication was observed in the lungs of all 3 ferrets infected with H5N1-ScrbT, whereas virus was detected in the lungs of only 1 out of 3 H5N1-126T infected ferrets (Fig 6C). Similar to our observations in the mouse model, H5N1-ScrbT showed higher levels of extrapulmonary replication (olfactory lobes and brain) as compared to H5N1-126T infected ferrets (Fig 6C). Unfortunately, due to the limitations of the ferret model, the small sample size did not provide enough statistical power to definitively demonstrate significance[45]. Taken together, these results indicate that endothelial cell tropism contributes to the enhanced virulence and systemic spread of H5N1 in ferrets.

### Multibasic cleavage site in HA is critical for endothelial cell tropism associated pathogenesis

**Fig 7:**
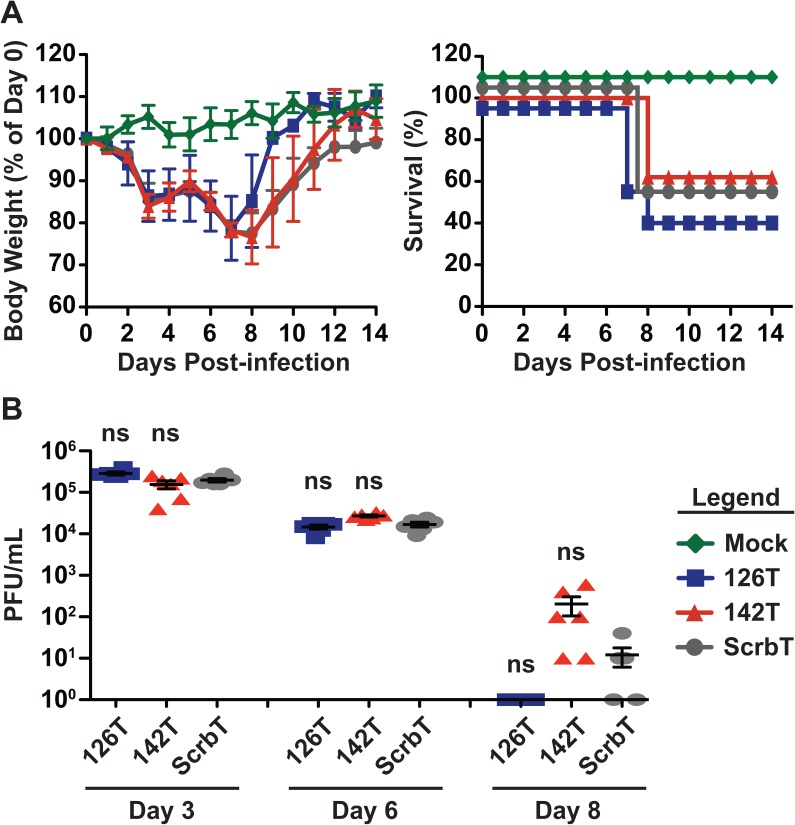
Restriction of low pathogenic H5N1 replication in endothelial cells does not alter disease outcome in mice. (A) Body weight loss and survival of infected mice. C57BL/6J mice (n = 5) were intranasally infected with 100 PFU of tropism restricted low pathogenic H5N1 viruses and monitored daily for body weight and survival. Left—Body weight loss, shown as relative percentage of day 0 weight (mean ± SEM) and Right–Survival. For better display, survival lines are offset by a few percentage points. (B) Viral titers in the lungs of infected mice. C57BL/6J mice (n = 5–6) were infected with 100 PFU and viral loads in the lungs on days 3, 6, and 8 pi were determined by plaque assay (PFU/mL). Each data point represents an individual mouse (mean ± SEM). Asterisk denotes statistical significance determined by one-way ANOVA in comparison to the H5N1-ScrbT group and the values are denoted as *p<0.05, **p<0.01, ***p<0.001 and ns–non significant. Data presented here is a representative of two independent experiments.

Prior studies have demonstrated that the MBS in HA is critical for the extrapulmonary spread of H5N1 viruses. The removal of the MBS in HA (low pathogenic) restricts H5N1 replication to the respiratory tract [41,46]; however, if the MBS also contributes to endothelial cell tropism mediated pathogenesis remains unknown. To address this, we generated low pathogenic H5N1 miR-targeted viruses with an MBS deleted HA (HA low pathogenic—HALo), and evaluated replication in cell culture and virulence in mice. As anticipated, miR-targeted H5N1 (HALo) viruses demonstrated cell type specific restriction of replication in human, mouse and ferret cell lines (S3 Fig, S4B Fig). Next, we investigated the pathogenesis of the HALo viruses in mice. C57BL/6J mice were intranasally infected with 100PFU of H5N1 (HALo) miR-targeted viruses and monitored for body weight loss and survival (Fig 7A). Interestingly, H5N1-126T (HALo) infected mice showed similar weight loss and survival as compared to the H5N1-ScrbT (HALo) and H5N1-142T (HALo) groups. In addition, we observed similar levels of virus replication in the lungs of infected mice for all three groups (Fig 7B). Importantly, these studies with HALo viruses rule out the possibility of endothelial cell independent attenuation of H5N1-126T viruses in mice. Taken together, these studies demonstrate that the MBS in HA is required for endothelial cell tropism mediated pathogenesis of H5N1 viruses.

## Discussion

HPAI H5N1 virus infection causes severe respiratory distress and can be fatal even in healthy adults. A combination of viral and host factors have been shown to contribute to the rapid progression of disease during H5N1 infection. Here, we investigated if the cell tropism of H5N1 virus is a determinant of pathogenesis by utilizing endogenous miRNA mediated restriction of viral tropism [27,28,29]. Our studies demonstrate that H5N1 virus infection of endothelial cells contributes to the high virulence observed in mammalian species. Restriction of H5N1 replication via endothelial cell specific miR-126 ameliorated disease symptoms, limited mortality, and prevented systemic viral spread as compared to a control virus in both mouse and ferret models. In addition, prevention of H5N1 replication in endothelial cells lowered the levels of inflammatory cytokines in the lungs and limited excessive vascular leakage. Taken together, our studies demonstrate the significance of endothelial cell tropism in the pathogenesis of H5N1 virus in mammalian species.

Several prior studies have utilized genetic reassortments of pathogenic and non-pathogenic strains to investigate the basis for the severe disease associated with H5N1 infections [47,48,49]. However, such comparisons of pathogenic versus non-pathogenic viruses are often associated with several log differences in viral loads in the lungs. As such, the increased virulence of H5N1 virus has been attributed to the higher levels of viral replication observed in these studies. We took a Complementary approach by utilizing isogenic HPAI H5N1 viruses differing in only the incorporated miRNA target sites. Interestingly, endothelial cell or hematopoietic cell restriction did not alter the peak viral loads in the lungs of mice, indicating a minimal contribution by these cell types to new virion production. However, it should be noted that the H5N1-126T infected mice showed lowered viral loads in the lungs on Day 2 pi. Prior studies suggest that lowered early viral titers can result in decreased virulence [50]. Importantly, as mice infected with tropism restricted H5N1 viruses display different disease outcomes, despite showing similar peak viral loads in the lungs, these results demonstrate that viral loads are not the sole determinant of H5N1 pathogenesis.

Avian influenza virus strains of the H5 and H7 subtypes can gain an MBS in the HA gene while circulating in terrestrial poultry. An MBS is one of the main determinants of pathogenesis for H5 and H7 strains in several mammalian and avian models, as the MBS allows for the processing of HA by ubiquitously expressed furin proteases and thus permits systemic viral replication [40,41,46]. As such, removal of the MBS in HA reduces virulence and restricts viral replication to the respiratory tract of infected mice and ferrets [41,46]. Our studies demonstrate that H5N1 infection of endothelial cells, which results in increased damage and vascular leakage in the lungs, is also a critical component of H5N1 pathogenesis, as H5N1-126T virus replication was restricted to the lungs in mice, despite containing an HA segment with an MBS. Moreover, in the ferret model, H5N1-126T virus was restricted to the upper respiratory tract, and failed to efficiently spread to the lower respiratory tract or to the olfactory bulb and brain. In both humans and ferrets, avian H5N1 viruses can replicate in the lower respiratory tract due to the abundance of alpha 2’-3’ linked sialic acid receptors [51,52]. It is interesting to note that H5N1-126T failed to replicate in the lungs of ferrets; this raises the possibility that H5N1 viral spread to the lower respiratory tract can occur via endothelial cell infection. In corroboration with this, the regions of infection and inflammation in the lungs of H5N1-126T infected mice were smaller as compared to the H5N1-ScrbT and H5N1-142T groups (Fig 4C). As the viral loads were similar in mouse lungs and ferret nasal washes of H5N1-126T and H5N1-ScrbT infected animals, the lack of systemic viral spread in H5N1-126T virus infected animals was not due to differences in viral replication. To further rule out the possibility of endothelial cell independent attenuation of miR-126T viruses, we generated H5N1 (HALo) miR-targeted viruses to restrict replication in airway epithelial cells and observed similar virulence for H5N1-126T (HALo) and H5N1-ScrbT (HALo) viruses. Taken together, our studies demonstrate that the MBS in HA is critical for endothelial cell infection induced vascular damage and subsequent systemic spread of H5N1 virus.

Robust HPAI H5N1 infection has been observed in the reticulo-endothelial system of avian models, and this endothelial tropism has been implicated in high virulence in avian hosts [24,25,53]. Until recently, endothelial cells were considered non-significant for the pathogenesis of H5N1 virus in mammalian species, as there are limited reports on H5N1 infection in endothelial cells of mammalian models [25,26]. Recent research has shed light on the role of endothelial cells in influenza virus pathogenesis in mammalian species, as studies now demonstrate that human endothelial cells are highly susceptible to H5N1 and H7N9 infection in vitro and are a source of proinflammatory cytokines in the lungs [34,54,55]. Through the use of endogenous miRNA-mediated restriction of viral replication, we show that H5N1 infection of endothelial cells is critical for elevated cytokine production in vivo. Importantly, our studies provide a new mechanism for H5N1 pathogenesis in mammalian species, whereby H5N1 infection of endothelial cells elevates cytokine levels and compromises endothelial barrier function in the lungs, ultimately resulting in increased vascular leakage and viral pneumonia.

Previous studies by us and others show that hematopoietic cells are susceptible to infection by influenza viruses in vivo [56,57,58]. In addition, in vitro studies demonstrate that influenza virus replication in hematopoietic cells is necessary for the activation of innate RNA sensors and for efficient cytokine production [28]. Upon infection with an H5N1 virus with restricted hematopoietic cell tropism (H5N1-142T), we observed lowered expression of inflammatory cytokines in the lungs as compared to H5N1-ScrbT infected mice. We also observed decreased microvascular leakage in the lungs of mice infected with H5N1-142T as compared to H5N1-ScrbT. However, vascular leakage in the lungs of H5N1-142T infected mice was higher in comparison to the H5N1-126T group, suggesting that both inflammatory cytokines and direct H5N1 infection of endothelial cells contribute to disruption of endothelial barrier integrity. These findings are in agreement with prior studies in which treatment with immunosuppressive drugs lowered cytokines levels yet did not affect mortality during H5N1 infection [59,60]. In contrast, treatments that strengthen endothelial barrier function improved survival of H5N1 infected mice [61]. Taken together, these studies suggest that immunosuppressive treatment alone may not be sufficient to treat H5N1 infection; rather, combination therapies that target viral components, suppress host immune responses, and strengthen endothelial barrier integrity may reduce H5N1 disease burden.

In conclusion, our study demonstrates that endothelial cell tropism is a determinant of the high virulence associated with HPAI H5N1 infection in mammalian hosts. By utilizing endogenous miRNA mediated restriction of viral tropism, we demonstrate that H5N1 infection of endothelial cells results in increased cytokine production in the lungs and loss of endothelial barrier function, which culminates in vascular leakage in the lungs. In addition, extrapulmonary spread of H5N1 virus likely occurs via the hematogenous route by infection of endothelial cells. Importantly, our studies strongly suggest that a combination therapy with drugs that improve endothelial barrier function and suppress host immune responses will be necessary for treating HPAI H5N1 virus infections. Apart from HPAI H5N1 virus, several other influenza virus strains either with (H7N7) or without (1918, H7N9) an MBS in HA have been shown to cause aggressive disease in mammalian hosts. Future studies are necessary to determine if the high virulence of other pathogenic influenza virus strains is due to endothelial cell tropism.

## Materials and methods

### Biosafety practices

Studies with highly pathogenic H5N1 viruses were performed in a CDC/USDA accredited ABSL3 laboratory by skilled researchers with more than 4 years of experience in the appropriate animal models.

### Ethics statement

All studies were performed in accordance with the principles described by the Animal Welfare Act and the National Institutes of Health guidelines for the care and use of laboratory animals in biomedical research. The protocols for performing mouse and ferret studies were reviewed and approved by the Institutional Animal Care and Use Committee (IACUC) at the University of Chicago and at the Icahn School of Medicine (ACUP 72279; IACUC-2013-1408).

### Mouse studies

Eight week old C57BL/6J female mice were purchased from Jackson Laboratory and housed in an Animal Biosafetly Level 3 (ABSL-3) facility. Mice were anesthetized with ketamine/xylazine and intranasally instilled with the indicated dose of virus diluted in 25μl PBS as previously described [62]. Infected animals were monitored daily for body weight loss and survival for 14 days. Animals showing weight loss of >25% or neurological symptoms of infection were considered to have reached the experimental end point and were humanely euthanized.

### Ferret studies

Five-month old male finch ferrets that tested seronegative for circulating influenza viruses were purchased from Triple F Farms and housed in a ABSL3 facility. Ferrets were anesthetized with ketamine HCl (30mg/Kg) and xylazine (2mg/Kg), and then intranasally infected with 3500 PFU of H5N1 virus diluted in PBS. Ferrets were monitored daily for body weight loss. The humane endpoints for euthanasia included >15% body weight loss or neurological complications.

### Cell lines

Human lung epithelial adenocarcinoma cells (A549, ATCC), human embryonic kidney cells (293T, ATCC), mouse lung epithelial cells (LA-4, ATCC) and mouse endothelial cells (MS1, ATCC) were maintained in DMEM (Gibco) supplemented with 10% fetal bovine serum (FBS, Denville Scientific) and penicillin/streptomycin (Pen/Strep, 100 units/mL, Corning). Human lung microvascular endothelial cells (HMVEC, Lonza) were maintained in EGM^TM^ -2 media according to the supplier’s instructions (Lonza). Madin-Darby Canine Kidney (MDCK, ATCC) cells were maintained in Minimum Essential Medium (MEM; Lonza) supplemented with 10% FBS and Pen/Strep (100 units/mL). THP-1 cells (ATCC) were maintained in RPMI media (Gibco) supplemented with 10% FBS and Pen/Strep (100 units/mL). Bone marrow derived dendritic cells (BMDC) were prepared from the tibia and femur of C57BL/6J mice.

### Generation of cell tropism restricted H5N1 viruses

Recombinant HPAI H5N1 viruses based on a human isolate (A/Vietnam/1203/2004 strain) were generated using the reverse genetics system [63,64,65]. The NP segments carrying miRNA target sites were generated as previously described [28]. Briefly, four copies of the miRNA target site (Complementary to the mature miRNA sequence) were incorporated into the 3’UTR of the NP gene immediately after the stop codon followed by duplication of the ~200nt NP segment packaging sequence. The Complementary sequences of miR-126-3p (126T; CGCATTATTACTCACGGTACGA), miR-142-3p (142T; TCCATAAAGTAGGAAACACTACA), and scrambled target sequence (ScrbT; GAGAATCTAAACGACTCAATACA) were incorporated into the NP segment. Recombinant H5N1-126T, H5N1-142T, H5N1-DblT and H5N1-ScrbT viruses were rescued as previously described [66]. Briefly, 0.5 μg of each of the eight pDZ plasmids representing the eight segments of the H5N1 genome were transfected into HEK-293T cells using Lipofectamine 2000 (Invitrogen), and was followed by co-culture with MDCK cells. After 48h post-transfection, ~200μl of the supernatants were transferred onto fresh MDCK cells seeded in 6 well plates and monitored for cytopathic effects. Recombinant viruses were plaque purified, propagated in MDCK cells, and the viral stocks were sequenced (Sanger sequencing) and confirmed to be free of unwanted mutations. Low pathogenic H5N1 (A/Vietnam/1203/2004) miR-targeted viruses were generated using an HA gene without the polybasic cleavage site [63].

### Western blot analysis

HEK-293T cells were co-transfected with a vector expressing a specific miRNA and a pDZ vector expressing an NP with miRNA target sites at a ratio of 8:1 using Lipofectamine 2000 reagent (Invitrogen). At 48h post-transfection, the cells were lysed with 1% NP40 lysis buffer and western blot analysis was performed using an NP specific monoclonal antibody (BEI Resources, #4554). The levels of Ku80 antigen were included as loading controls (Sigma).

### Infection assays in cell culture

A549 (4x10^5^/well), HMVEC (1x10^5^/well), THP-1 (4x10^5^/well), LA-4 (4x10^5^/well), J774 (4x10^5^/well) and MS1 (4x10^5^/well) cells were seeded in 12-well plates a day prior to infection. Cells were infected at the indicated MOI calculated based on cell numbers determined prior to infection. Infections were carried out in DMEM/0.2% BSA media for the indicated times and viral titers in the supernatants were determined by plaque assay on MDCK cells. BMDCs (1x10^6^/well) were seeded in 12-well plates, infected at an MOI = 1, and at 24hpi, the levels of infection were determined by flow cytometry using an anti-H5 monoclonal antibody (BEI Resources). Low pathogenic virus replication assays were performed in the presence of 1μg/ml of TPCK-treated trypsin (Sigma).

### Plaque assay

Viral titers in the cell culture supernatants, tissue homogenates, and nasal washes were determined by plaque assay on MDCK cells. Tissues from infected mice were harvested at the indicated dpi and homogenized in 1mL of PBS/0.2% BSA (MP Biomedicals) and clarified of debris by centrifugation. Serially diluted supernatants were added to confluent monolayers of MDCK cells seeded in 12-well plates and overlayed with MEM media (1x MEM with 0.2% BSA, Avicel (FMC Biopolymers), 0.1% NaHCO3 (Sigma), 0.01% DEAE-dextran (MP Biomedicals)). After a 48h incubation at 37°C, the cells were fixed with 4% formaldehyde for 1h and plaques were visualized by staining with 0.1% crystal violet solution. Plaque assays for low pathogenic viruses were performed in the presence of 1μg/ml of TPCK-treated trypsin (Sigma).

### Flow cytometric analysis of H5N1 infection in endothelial cells

Single cell suspensions of mouse lungs were prepared as described previously [58]. Briefly, lungs from naïve or infected mice were harvested, chopped into fine pieces with scissors and digested in HBSS buffer (Lonza) supplemented with 10% FBS and 0.4mg/ml collagenase (Sigma) at 37°C for 45 min. To achieve single cell suspensions, lung homogenates were passed through a 19G blunt needle and filtered through a 70μm cell strainer. Cell pellets were washed with FACS buffer (PBS, 1% FBS, 2mM EDTA) and subjected to RBC lysis (Biowhitaker) followed by two washes with FACS buffer. Single cell preparations were incubated in FACS buffer containing a mixture of 10μg/mL Fc receptor block, anti-CD45 (2μg/mL, clone 30-F11; Biolegend), anti-CD31 (2μg/mL; clone MEC 13.3; Biolegend), and biotinylated anti-H5 monoclonal antibody (1C10 clone, BEI resources), and followed by staining with Streptavidin-BV605 (Biolegend). Live/dead cells were separated using a Live/Dead fixable near IR staining kit (Life Technologies). After staining, samples were fixed with 0.1% formaldehyde in PBS and analyzed using a BD ARIA II housed inside a biosafety cabinet. Data analysis was performed using FlowJo software (Treestar Corp). Infected endothelial cells were identified using cells surface markers (CD45^-^, CD31^+^ and viral HA^+^).

### Quantitative RT-PCR analysis

Total RNA from the lungs of naïve or infected mice were prepared by homogenizing the entire lung in 1mL Trizol (Life technologies) and followed by extraction using the manufacturer’s recommendations. cDNA was generated by SuperScript II using oligo dT primers (Invitrogen). Real-time PCR was performed on an ABI7300 real time PCR system using SYBR Green (ABI Biosystems). Delta delta cycle thresholds were calculated using tubulin as the endogenous housekeeping gene and data is presented as fold change in expression over naïve mice. Primers used for qPCR analysis have been previously described [62].

### Evans Blue assay

C57BL/6J mice were infected with 25PFU of H5N1 virus. On day 7 pi mice were anesthetized with ketamine/xylazine and injected with Evans Blue dye (30mg/kg; Sigma) via retro-orbital route. After 1h, mice were euthanized and bronchioalveolar fluid (BALF) was collected using 1mL of PBS. The levels of Evans Blue dye were determined using a previously established protocol [39]. Briefly, BALF was mixed with formamide at a 1:4 ratio, mixed well by vortexing, and incubated for 18 h at 60°C. At the end of the incubation, the mixture was centrifuged at 3000 rpm for 30 min. Evans Blue dye levels in the supernatant were quantified by measuring the absorbance at 620 nm and 740 nm using a BioMate3 Spectrophotometer (Thermo) and the concentrations were determined with a standard curve.

### Histology

Mouse lungs were injected with 1mL of 4% formaldehyde through the trachea and tied-off with suture thread, and kept in formaldehyde for 2 days. Subsequently the lungs were embedded in paraffin, sectioned into 5μM slices and stained with hematoxylin and eosin. Slides were blinded and analyzed by a veterinary pathologist under a microscope.

### Statistical analysis

Data were analyzed using Prism GraphPad software and statistical significance was determined by one-way ANOVA. Asterisks *, **, and *** denote a significance with p-Values <0.05, 0.01, and 0.001 respectively.

## Supporting information

S1 Table Conservation of miR-126-3p and miR-142-3p among different species. (PDF)

S1 Fig Analysis of replication kinetics of H5N1 miRNA targeted viruses. Human and mouse cell lines were infected at the indicated MOI and at various times post-infection the supernatants were collected and titers were determined by plaque assay on MDCK cells. The titers are shown as PFU/mL (mean ± SEM). The limit of detection is 10 PFU/mL. The cell lines were infected at MOIs: A549 (0.001), THP-1 (0.01), HMVEC (0.01), LA-4 (1), J774 (0.01), and MS1 (1).
(TIF)

S2 Fig Comparison of virulence of wildtype H5N1 virus to H5N1-ScrbT in mice. C57BL/6J mice (n = 5) were intranasally infected at a dose of 25 PFU and monitored daily for weight loss and survival. Left—Body weight loss, shown as relative percentage of day 0 weight (mean ± SEM) and Right–Survival.
(TIF)

S3 Fig Analysis of replication kinetics of H5N1 miRNA targeted viruses in ferret lung epithelial cells. Ferret cells were infected at an MOI = 0.001 and at various times post-infection supernatants were collected, and titers were determined by plaque assay on MDCK cells. Left–H5N1 viruses with an HA containing the multibasic cleavage site (High Path). Right–H5N1 viruses with an HA lacking the multibasic cleavage site (Low Path).
(TIF)

S4 Fig Analysis of replication kinetics of low pathogenic H5N1 miRNA-targeted viruses. Human and mouse cell lines were infected at the indicated MOI and at various times post-infection supernatants were collected for viral titer determination. The titers are shown as PFU/mL (mean ± SEM). The limit of detection is 10 PFU/mL. The cell lines were infected at MOIs: A549 (0.001), THP-1 (0.01), HMVEC (0.01), LA-4 (1), J774 (0.01), and MS1 (1).
(TIF)
