# Supplementary material for: Endothelial cell tropism is a determinant of H5N1 pathogenesis in mammalian species
Source: PLoS Pathog. 2017 Mar 10;13(3):e1006270. doi: 10.1371/journal.ppat.1006270 (PMC5362246; doi:10.1371/journal.ppat.1006270)
Supplement: S1 Table — (PDF) [file ppat.1006270.s001.pdf]

Supplementary Table 1: Conservation of miR-126-3p and miR-142-3p among different species

**Conservation of miR-126-3p**		
Human<br>NR_029695.1	**ucguaccgugaguaauaaugcg**	hsa-miR-126-3p
Mouse<br>NR_029541.1	**ucguaccgugaguaauaaugcg**	mmu-miR-126a-3p
Ferret Genome	**ucguaccgugaguaauaaugcg**	Putative miRNA<br>(not annotated)
Chicken<br>NR_031468	**ucguaccgugaguaauaaugcg**	gga-miR-126-3p

**Conservation of miR-142-3p**		
Human<br>NR_029683	**uguaguguuuccuacuuuaugga**	hsa-miR-142-3p
Mouse<br>NR_029555.1	**uguaguguuuccuacuuuaugga**	mmu-miR-142a-3p
Ferret Genome	**uguaguguuuccuacuuuaugga**	Putative miRNA<br>(not annotated)
Chicken<br>NR_031503.1	**uguaguguuuccuacuuuaugga**	gga-miR-126-3p
