# Supplementary figures and images for: Endothelial cell tropism is a determinant of H5N1 pathogenesis in mammalian species
Source: PLoS Pathog. 2017 Mar 10;13(3):e1006270. doi: 10.1371/journal.ppat.1006270 (PMC5362246; doi:10.1371/journal.ppat.1006270)

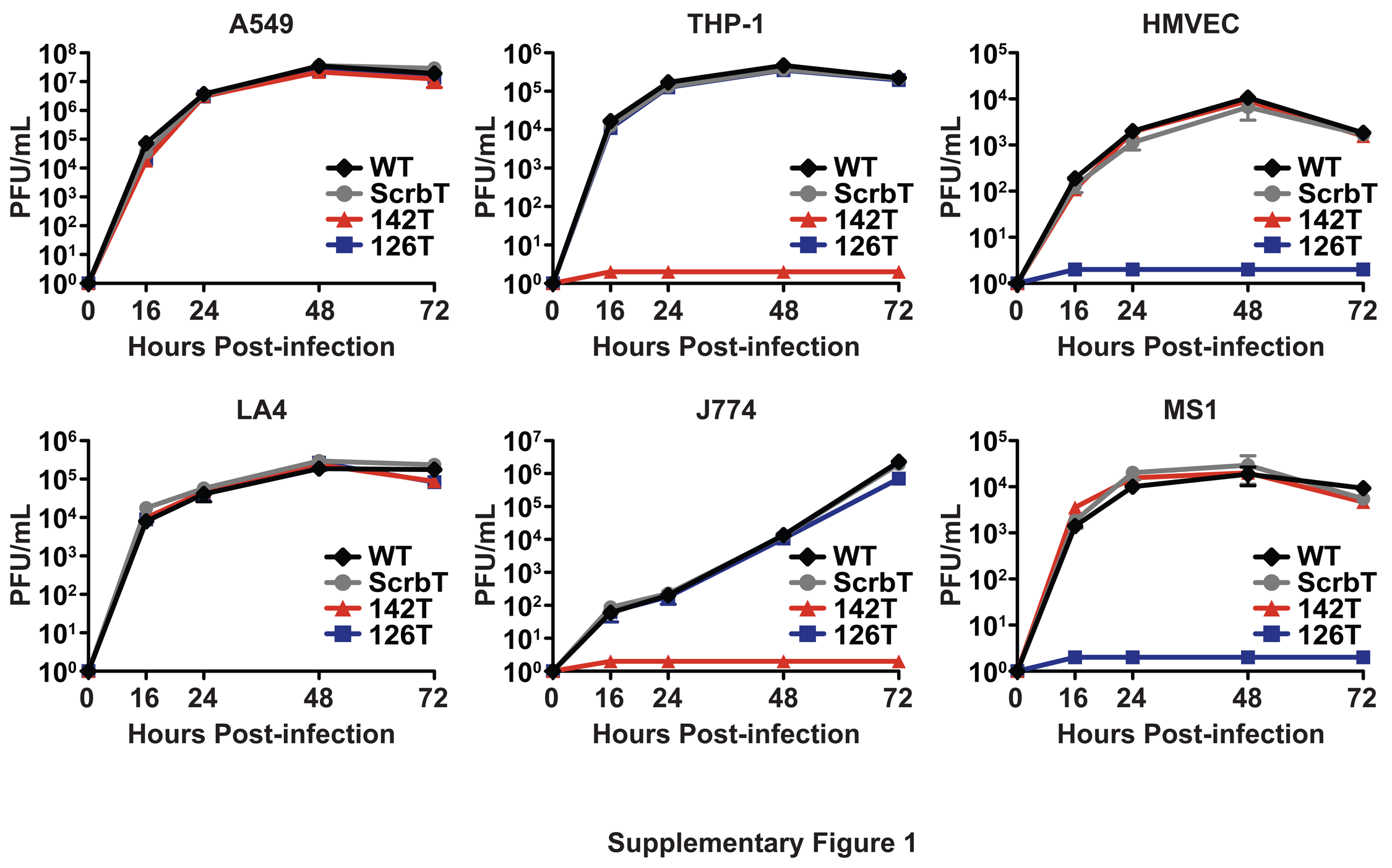

Supplement: S1 Fig — Human and mouse cell lines were infected at the indicated MOI and at various times post-infection the supernatants were collected and titers were determined by plaque assay on MDCK cells. The titers are shown as PFU/mL (mean ± SEM). The limit of detection is 10 PFU/mL. The cell lines were infected at MOIs: A549 (0.001), THP-1 (0.01), HMVEC (0.01), LA-4 (1), J774 (0.01), and MS1 (1). (TIF) [file ppat.1006270.s002.tif]

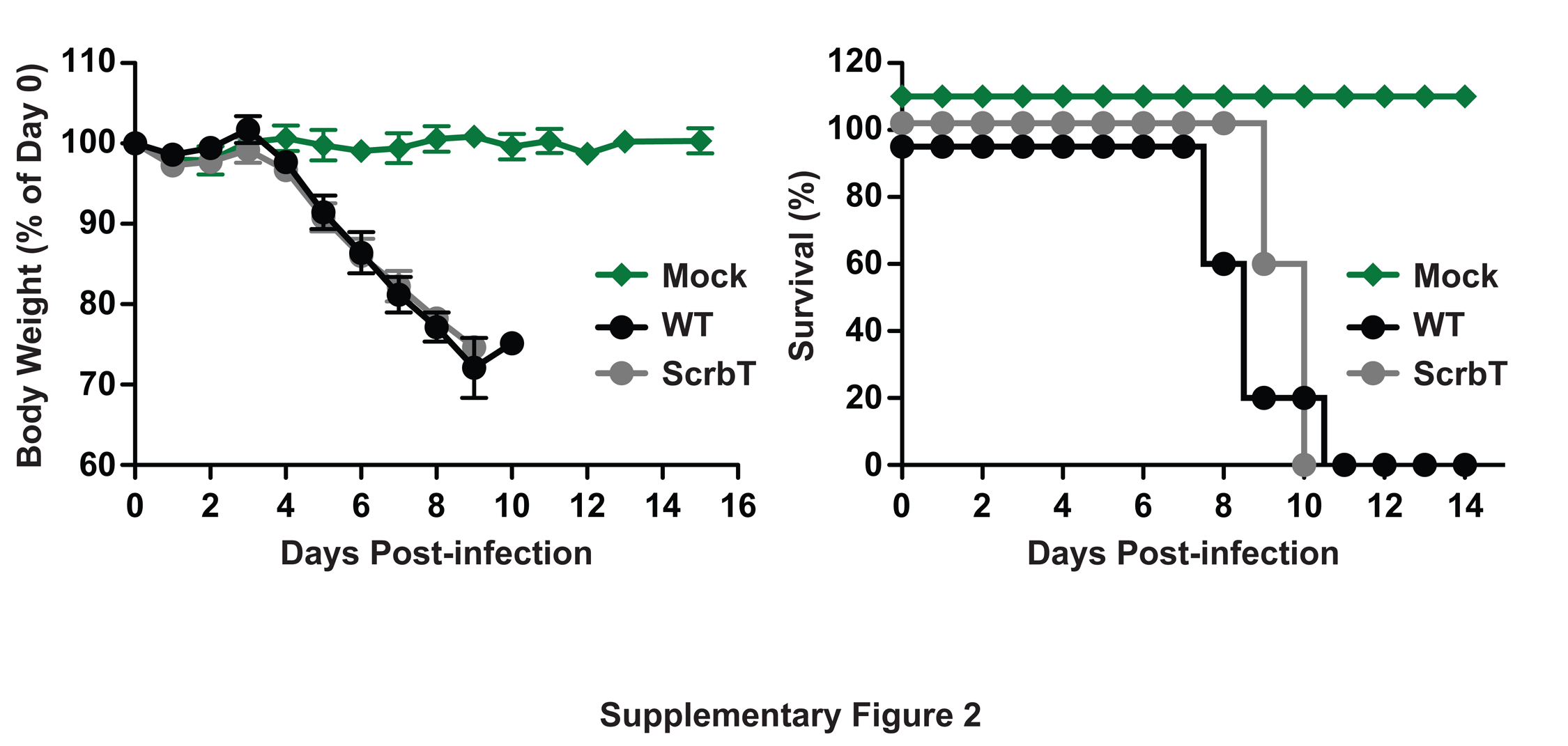

Supplement: S2 Fig — C57BL/6J mice (n = 5) were intranasally infected at a dose of 25 PFU and monitored daily for weight loss and survival. Left—Body weight loss, shown as relative percentage of day 0 weight (mean ± SEM) and Right–Survival. (TIF) [file ppat.1006270.s003.tif]

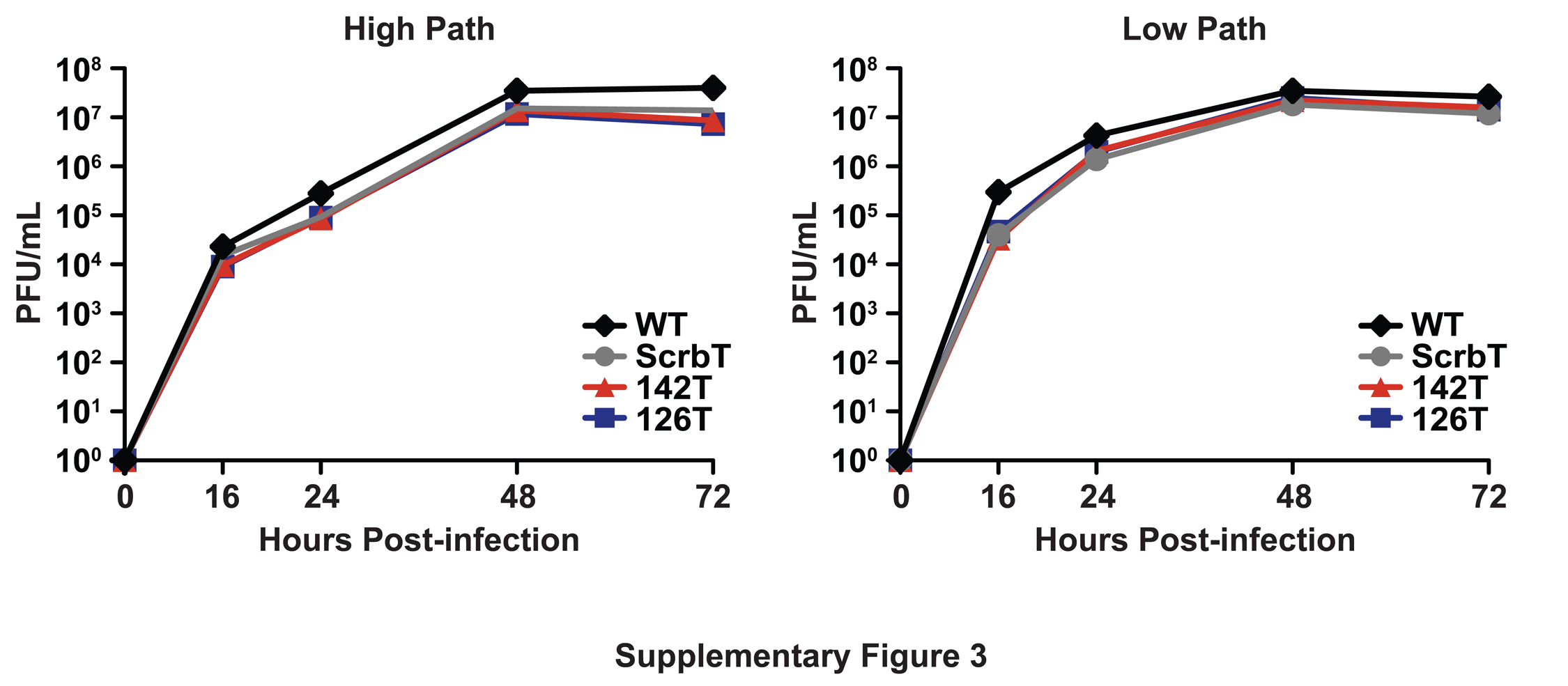

Supplement: S3 Fig — Ferret cells were infected at an MOI = 0.001 and at various times post-infection supernatants were collected, and titers were determined by plaque assay on MDCK cells. Left–H5N1 viruses with an HA containing the multibasic cleavage site (High Path). Right–H5N1 viruses with an HA lacking the multibasic cleavage site (Low Path). (TIF) [file ppat.1006270.s004.tif]

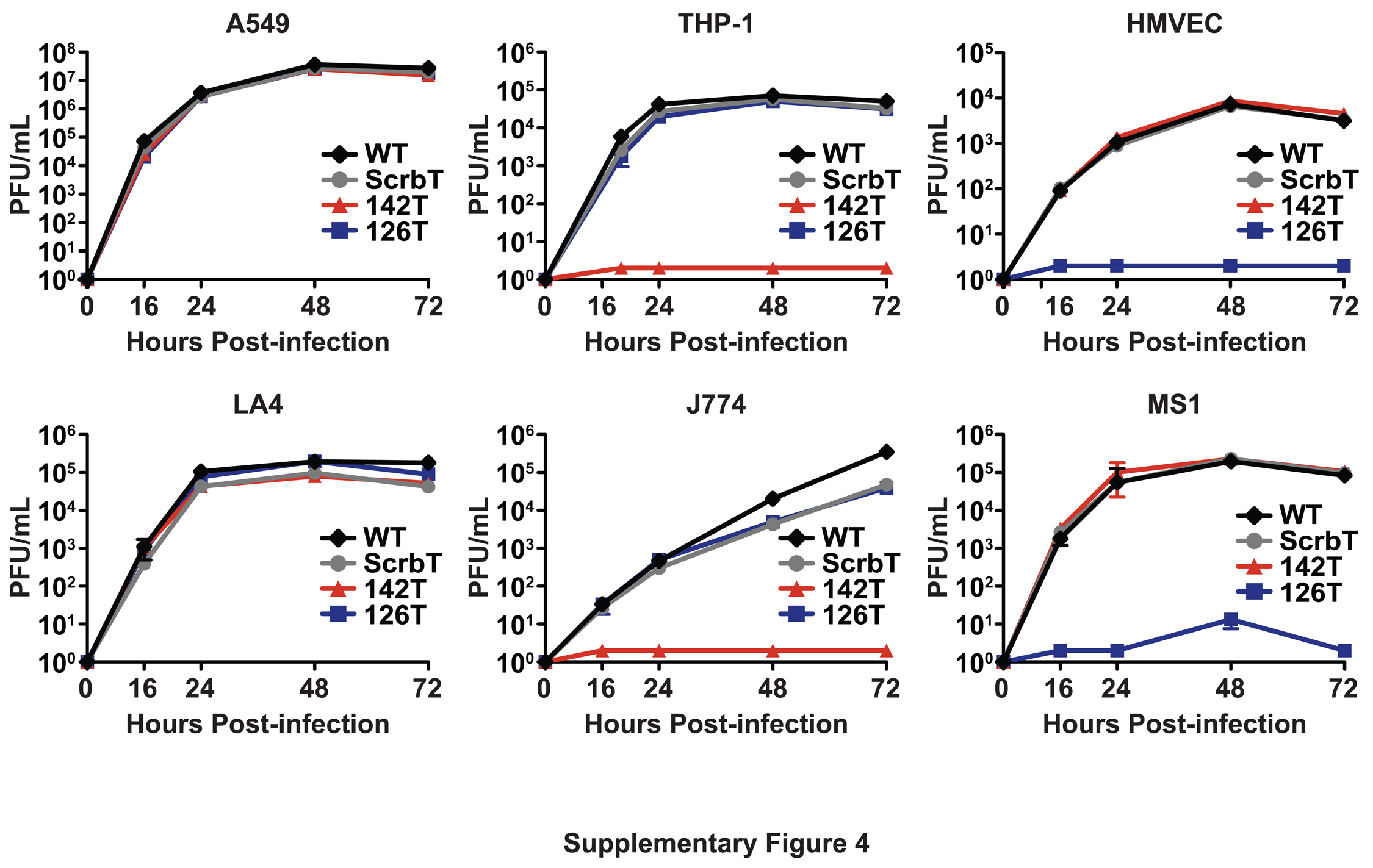

Supplement: S4 Fig — Human and mouse cell lines were infected at the indicated MOI and at various times post-infection supernatants were collected for viral titer determination. The titers are shown as PFU/mL (mean ± SEM). The limit of detection is 10 PFU/mL. The cell lines were infected at MOIs: A549 (0.001), THP-1 (0.01), HMVEC (0.01), LA-4 (1), J774 (0.01), and MS1 (1). (TIF) [file ppat.1006270.s005.tif]
